# Antimicrobial and Antioxidant Activities of the Root Bark Essential Oil of *Periploca sepium* and Its Main Component 2-Hydroxy-4-methoxybenzaldehyde

**DOI:** 10.3390/molecules15085807

**Published:** 2010-08-24

**Authors:** Jihua Wang, Hao Liu, Jianglin Zhao, Haifeng Gao, Ligang Zhou, Zhilong Liu, Yuanquan Chen, Peng Sui

**Affiliations:** College of Agronomy and Biotechnology, China Agricultural University, Beijing 100193, China

**Keywords:** Asclepiadaceae, Apocynaceae, *Periploca sepium*, essential oil, 2-hydroxy-4-methoxybenzaldehyde, antimicrobial activity, antioxidant activity

## Abstract

The root bark essential oil of *Periploca sepium* Bunge (Asclepiadaceae/ Apocynaceae) obtained by hydrodistillation was investigated by GC and GC-MS. 2-Hydroxy-4-methoxybenzaldehyde was found to be the main component (78.8% of the total) among 17 identified compounds. 2-Hydroxy-4-methoxybenzaldehyde was separated and purified by preparative thin layer chromatography, and was further identified by means of physicochemical and spectrometric analysis. Both the essential oil and 2-hydroxy-4-methoxybenzaldehyde exhibited antimicrobial activities on all test bacteria and fungi, with MBC/MFC values ranging from 125 μg/mL to 300 μg/mL, MIC values from 80 μg/mL to 300 μg/mL, and IC_50_ values from 63.29 μg/mL to 167.30 μg/mL. They also showed moderate antioxidant activity in the DPPH free radical scavenging, β-carotene bleaching and ferrozine-ferrous ions assays. The results indicated that the major portion of these antimicrobial and antioxidant activities was due to the presence of 2-hydroxy-4-methoxy-benzaldehyde in the oil, which could potentially be developed as an antimicrobial and antioxidant agent in the future.

## 1. Introduction

In recent years, due to the growing consumer demand for food products free of synthetic chemical additives increasing attention has been paid to the exploration of naturally-occurring antioxidants and antimicrobials [1,2]. The plant kingdom, with its remarkable diversity of natural compounds has merited special interest. Plant essential oils and their components have been shown to possess multiple and varied biological activities such as antimicrobial, insecticidal and antioxidant properties [3] and they have received much attention to prevent plant and animal diseases as well as to prevent oxidative damage [4,5].

*Periploca sepium* Bunge belongs to the Asclepiadaceae or Apocynaceae family and is widely distributed in China [6]. The root barks of *P. sepium* have been used as a Traditional Chinese Medicine for the treatment of rheumatoid arthritis and wounds. However, toxicity at high-doses was often observed due to the presence of cardiac glycosides [7]. Previous phytochemical studies on *P. sepium* resulted in the identification of pregnane glycosides, cardiac glycosides, oligosaccharides, coumarins, flavonoids and triterpenoids [8]. To the best of our knowledge, the antimicrobial and antioxidant activities of the essential oil of *P. sepium* have not been studied previously, although there were some reports clarifying its chemical composition as well as its insecticidal activity [9,10]. The aim of the present study was to analyze the chemical composition of the root bark essential oil of *P. sepium* from China as well as to evaluate its antimicrobial and antioxidant activities.

## 2. Results and Discussion

### 2.1. Essential oil analysis

The essential oil was obtained in 0.15% yield by hydrodistillation of the root bark of *P. sepium*. The chemical composition was analyzed by GC and GC-MS. Seventeen compounds were identified, which accounted for 95.8% of the total oil (Table 1). Phenolics, monoterpenoids, and aliphatics in the oil accounted for 80.1%, 12.4% and 3.3%, respectively. The major compounds in the oil were 2-hydroxy-4-methoxybenzaldehyde (78.8%), α-terpineol (2.6%), β-thujene (2.3%), linalool (2.2%), and β-myrcene (2.0%). The chemical profile of the root bark essential oil of *P. sepium* in this study was different from that of the previous reports [9,10], though the main compound with the highest content was 2-hydroxy-4-methoxybenzaldehyde. The reason could be attributed to the different geographical environment, cultivar type, growth season and physiological age of the plant besides the method of oil isolation [11].

The 2-hydroxy-4-methoxybenzaldehyde was further separated and purified by column chromatography and preparative thin layer chromatography as a colorless solid with a 39–40 °C melting point and it was characterized from its ^1^H-, ^13^C-NMR and mass spectra. After comparing the physicochemical and spectrometric data with those reported in the literature [12,13], the compound was further verified as 2-hydroxy-4-methoxy benzaldehyde.

**Table 1:** Chemical composition of the essential oil from the root bark of *P. sepium*.

Compound^a^	RI^b^	RA (%)^c^
β-Myrcene	950	2.0
β-Thujene	991	2.3
β-(*Z*)-Ocimene	1012	0.6
Linalool	1075	2.2
Borneol	1154	0.8
Terpinen-4-ol	1166	1.1
α-Terpineol	1181	2.6
Bornyl acetate	1286	0.8
2-Hydroxy-4-methoxybenzaldehyde	1352	78.8
Paeonol	1459	0.6
Vanillin	1477	0.3
Methyl 4-methoxysalicylate	1483	0.4
*n*-Hexadecoic acid	1960	0.8
Ethyl palmitate	2017	0.6
α-Linoleic acid	2082	0.6
Ethyl linolate	2090	0.5
Ethyl oleate	2092	0.8
Total identified		95.8
Monoterpenoids		12.4
Phenolics		80.1
Aliphatics		3.3

^a^: The identified constituents are listed in their order of elution. ^b^: RI indicates the retention indices calculated against C_8_-C_40_ *n*-alkanes on the HP-5MS column. ^c^: RA indicates relative amount (peak area relative to the total peak area).

### 2.2. Antimicrobial activity

The antimicrobial activities of the essential oil and 2-hydroxy-4-methoxybenzaldehyde were evaluated against 10 test microorganisms, including five Gram-negative bacteria, three Gram-positive bacteria, and two fungi. Their potency was assessed quantitatively by MBC, MIC and IC_50_ values which are shown in Table 2. The MIC values of the oil on test microorganisms ranged from 100 μg/mL to 300 μg/mL, MBC values from 150 μg/mL to 300 μg/mL, and IC_50_ values from 84.12 μg/mL to 167.30 μg/mL. Of them, both *A. tumefaciens* and *S. aureus* were the most sensitive bacteria, with IC_50_ values of 99.09 μg/mL and 84.12 μg/mL, respectively. Correspondingly, the MIC values of 2-hydroxy-4-methoxybenzaldehyde on test microorganisms ranged from 80 μg/mL to 250 μg/mL, MBC values from 125 μg/mL to 250 μg/mL, and IC_50_ values from 63.29 μg/mL to 161.90 μg/mL. The fungus *C. albicans* used was possibly an amphotericin-resistant strain as it was not sensitive to the positive control amphotericin B, with the IC_50_ value of 713.13 μg/mL. The IC_50_ values of the oil and 2-hydroxy-4-methoxybenzaldehyde on *C. albicans* were 114.36 μg/mL and 99.99 μg/mL respectively, which indicated that they might be used as alternative antimicrobials on antibiotic-resistant *C. albicans*. The antimicrobial activity of 2-hydroxy-4-methoxybenzaldehyde was a little stronger than that of the essential oil, suggesting that 2-hydroxy-4-methoxybenzaldehyde was the main antimicrobial component in the root bark oil. This is the first report on the antimicrobial activity of 2-hydroxy-4-methoxybenzaldehyde though its antimicrobial mechanisms need to be further studied.

**Table 2:** Antimicrobial activity of *P. sepium* essential oil and 2-hydroxy-4-methoxy benzadehyde.

Test microorganism	*P. sepium *oil (μg/mL)			2-Hydroxy-4-methoxy- benzaldehyde (μg/mL)			Positive control^a^ (μg/mL)
	MBC/MFC	MIC	IC_50_	MBC/MFC	MIC	IC_50_	IC_50_
*A. tumefaciens*	200	125	99.09±0.29^b^	125	80	63.49±0.41	8.34±0.09
*E. coli*	200	200	144.84±1.34	200	200	101.88±1.82	10.47±0.31
*P. lachrymans*	250	200	167.30±0.69	200	200	141.74±0.55	9.01±0.09
*S. typhimurium*	200	150	105.99±0.93	200	150	95.05±1.20	91.46±0.55
*X. vesicatoria*	250	200	151.24±1.96	200	150	131.86±1.57	11.62±0.19
*B. subtilis*	200	200	136.63±1.78	200	150	111.49±0.24	4.98±0.06
*S. aureus*	150	100	84.12±0.61	150	80	63.29±0.38	78.60±0.61
*S. haemolyticus*	250	200	134.49±2.04	150	125	78.74±0.68	7.75±0.16
*C. albicans*	250	150	114.36±2.37	200	150	99.99±1.74	713.13±1.49
*M. oryzae*	300	300	152.52±2.77	250	250	161.90±1.60	38.44±0.56

^a^ : The positive controls for bacteria, *Candida albicans* and *Magnaporthe oryzae* were streptomycin sulfate, amphotericin B and carbendazim, respectively. ^b^ : Mean ± standard deviation for triplicate experiments.

### 2.3. Antioxidant activity

The root bark essential oil and 2-hydroxy-4-methoxybenzaldehyde were subjected to a screening for antioxidant activity by three complementary tests, namely the DPPH free radical scavenging, β-carotene-linoleic acid and ferrozine-Fe^2+^ complex formation systems [14]. The antioxidant activity results of the two samples along with the positive controls are shown in Table 3. DPPH is a stable free radical which can readily undergo reduction in the presence of an antioxidant. The ability of the samples to scavenge DPPH radical was determined on the basis of their concentrations, with IC_50_ values of 8.56 mg/mL for *P. sepium* oil, and 9.04 mg/mL for 2-hydroxy-4-methoxybenzaldehyde, respectively. The DPPH radical scavenging capability of 2-hydroxy-4-methoxybenzaldehyde was little weaker than that of the essential oil, so it is possible that the other compounds in the oil also contributed to the antiradical activity.

The β-carotene bleaching assay is based on the loss of the yellow color of β-carotene due to its reaction with radicals formed by linoleic acid oxidation in an emulsion. The rate of β-carotene bleaching can be slowed down in the presence of antioxidants [14]. The two samples inhibited β-carotene bleaching in a dose dependent variable with the IC_50_ values of 0.37 mg/mL for *P. sepium* oil and 0.25 mg/mL for 2-hydroxy-4-methoxybenzaldehyde, respectively (Table 3). It was speculated that 2-hydroxy-4-methoxy benzaldehyde quenched β-carotene oxidation effectively.

Chelating activity of the essential oil was determined by the ferrozine assay [15]. Ferrozine can quantitatively form complexes with Fe^2+^. In the presence of other chelating agents, the complex formation is disrupted with the result that the red color of the complex is decreased. Measurement of the rate of red color reduction therefore allows estimation of the chelating activity of the coexisting chelator. The two samples inhibited the formation of ferrozine-Fe^2+^ complex in a dose dependent manner with the IC_50_ values of 2.11 mg/mL for *P. sepium* oil, and 2.31 mg/mL for 2-hydroxy-4-methoxybenzaldehyde, respectively. As shown in Table 3, the chelating activity of 2-hydroxy-4-methoxybenzaldehyde was equivalent to that of the essential oil which indicated that 2-hydroxy-4-methoxy benzaldehyde was the main antioxidant component in the oil. 

Murthy *et al*. [16] previously reported the antioxidant activity of the root extract from *Decalepis hamiltonii* (Asclepiadaceae), and also found that the main antioxidant component in the extract was 2-hydroxy-4-methoxybenzaldehyde by using DPPH free radical scavenging and β-carotene bleaching assays.

**Table 3:** Antioxidant activity of *P. sepium* essential oil and 2-hydroxy-4-methoxy benzadehyde.

Assay	IC_50_ (mg/mL)		
	*P. sepium* oil	2-Hydroxy-4-methoxy- benzaldehyde	Positive control^a^
DPPH inhibition	8.56 ± 0.14^b^	9.04 ± 0.09	0.0257 ± 0.0004
β-Carotene bleaching	0.37 ± 0.01	0.25 ± 0.01	0.0315 ± 0.0007
Ferrozine-Fe^2+^ complex formation	2.11 ± 0.05	2.31 ± 0.02	0.0185 ± 0.0001

^a^: The positive controls for DPPH inhibition, β-carotene-linoleic acid and ferrous ions assays were BHT, BHT and EDTA, respectively. ^b^: Mean ± standard deviation for triplicate experiments.

## 3. Experimental

### 3.1. General

Silica gel (100-200 and 200-300 mesh, Qingdao Marine Chemical Company, China) was used for column chromatography (CC). Thin layer chromatography (TLC) and preparative thin layer chromatography (PTLC) plates were coated with 0.5-mm and 1-mm layers of silica gel (GF_254_, 300-400 mesh), respectively (Qingdao Marine Chemical Company, China). Melting point was determined on an XT4-100B microscopic melting-point apparatus (Tianjin Tianguang Optical Instruments Company, China) and uncorrected. NMR spectra were recorded on a Bruker-ARX-300 spectrometer (^1^H at 300 MHz and ^13^C at 75 MHz). ESI-MS spectra were recorded on a Bruker Esquire 6000 LC/MS spectrometer. The microplate spectrophotometer (PowerWave HT, BioTek Instruments, USA) was employed to measure the light absorption value. β-Carotene, carbendazim, streptomycin sulfate, 1,1-diphenyl-2-picrylhydrazyl (DPPH) and C_8_-C_40_ *n*-alkanes were purchased from Sigma-Aldrich (USA). Linoleic acid and ferrozine disodium salt were obtained from Johnson Matthey (UK). Amphotericin B and 3-(4,5-dimethylthiazol-2-yl)-2,5-diphenyl tetrazolium bromide (MTT) were purchased from Amresco (USA). Butylated hydroxytoluene (BHT), ferrous chloride (FeCl_2_), Tween-40, ethylene diamine tetraacetic acid (EDTA) were bought from Beijing Chemical Company. All other unlabelled chemicals and reagents were of analytical grade.

### 3.2. Plant material

The roots of *P. sepium* were collected in Hunan Province of China in March 2008. The root barks were dried in the shade at room temperature. The taxonomical identification of the plant material was done by Prof. Quanru Liu of Beijing Normal University. The voucher specimen of this collection (BSMPMI-200803001) was deposited at the Herbarium of the Institute of Chinese Medicinal Materials, China Agricultural University.

### 3.3. Isolation of the essential oil

The dry root barks (1 kg) of *P. sepium* were submitted to hydrodistillation in a Clevenger-type apparatus at 100 °C for 4 h. The distilled oil was extracted with diethyl ether and dried over anhydrous sodium sulfate. After filtration, the yield of the essential oil was 1.5 g (0.15%, w/w). It was then preserved in a sealed dark glass vial at 4 °C until required.

### 3.4. Purification and characterization of 2-hydroxy-4-methoxybenzaldehyde

The crude essential oil was chromatographed on a silica gel column by gradient elution with *n*-hexane first, then with *n*-hexane-ethyl acetate, and last with acetone to obtain 28 fractions. Of them, fraction 8 was further separated by PTLC with petroleum ether-acetone (10:1, v/v) to afford a pure compound. ^1^H-NMR (CDCl_3_, 300 MHz) *δ* (ppm): 11.48 (s, 1H, -OH), 9.72 (s, 1H, H-7), 7.42 (d, 1H, *J* = 8.4 Hz, Ph–H), 6.53 (dd, 1H, *J* = 8.4, 2.4 Hz, Ph–H), 6.43 (d, 1H, *J* = 2.4 Hz, Ph–H), 3.86 (s, 3H, -OCH_3_)]; ^13^C-NMR (CDCl_3_, 75 MHz) *δ* (ppm): 194.3 (C-7), 166.8 (C-4), 164.5 (C-2), 135.2 (C-1), 115.2 (C-5), 108.3 (C-6), 100.7 (C-3), 55.7 (C-8)]. Its identity was confirmed by the ESI-MS with the mass spectral fragmentation pattern [*m/z* (% abundance): 153(8), 152 (94), 151 (100), 123 (14), 109 (18), 81 (27), 53 (14)].

### 3.5. Analysis of essential oil

The composition of the essential oil of *P. sepium* was determined by the use of analytical GC (FID) and GC/MS techniques. The same column and analysis conditions were used for both GC and GC/MS. An Agilent 6890N Network GC system for gas chromatography was equipped with an HP-5MS column [30 m × 0.25 mm (5%-phenyl)-methylpolysiloxane capillary column, film thickness 0.25 µm], a split-splitless injector heated at 250 °C and a flame ionization detector (FID) at 240 °C. The oven temperature was programmed as follows: initial temperature 50 °C for 1.5 min, increase 10 °C/min up to 180 °C, 2 min at 180 °C, and then increase by 6 °C/min up to 280 °C, 10 min at 280 °C. Helium (99.999%) was used as carrier gas at a flow rate of 1.0 mL/min. The injection volume was 1.0 μL (split ratio 1:20). GC/MS analyses were performed using an Agilent 6890N Network GC system with an Agilent 5973 Network mass selective detector, mass spectrometer in EI mode at 70 eV in m/e range 10-550 amu. The components were identified by comparison of their mass spectra with the NIST 2002 library data of the GC-MS system, as well as by comparison of their retention indices (RI) with the relevant literature data [9,10,17,18]. The relative amount (RA) of each individual component of the essential oil was expressed as the percentage of the peak area relative to the total peak area. RI value of each component was determined relative to the retention times (RT) of a series of C_8_-C_40_ *n*-alkanes with linear interpolation on the HP-5MS column [19].

### 3.6. Antimicrobial activity

#### 3.6.1. Antibacterial activity assay

Three Gram-positive (*Bacillus subtilis* ATCC 11562, *Staphylococcus aureus* ATCC 6538 and *Staphylococcus haemolyticus* ATCC 29970), and five Gram-negative (*Agrobacterium tumefaciens* ATCC 11158, *Escherichia coli* ATCC 29425, *Pseudomonas lachrymans* ATCC 11921, *Salmonella typhimurium* ATCC 14028 and *Xanthomonas vesicatoria* ATCC 11633) bacteria were selected for antibacterial activity assay. They were grown in liquid LB medium (yeast extract 5 g/L, peptone 10 g/L, NaCl 5 g/L, pH 7.0) overnight at 28 °C, and the diluted bacterial suspension (10^6^ cfu/mL) was ready for detection. A modified broth dilution-colorimetric assay by using the chromogenic reagent 3-(4,5-dimethylthiazol-2-yl)-2,5-diphenyltetrazolium bromide (MTT) was used to detect the antibacterial activity of either the essential oil or 2-hydroxy-4-methoxybenzaldehyde [20]. Briefly, the sample was dissolved in acetone at an initial concentration of 10 mg/mL. Then it was diluted with 30% acetone to obtain concentrations ranging from 0.25 mg/mL to 3.0 mg/mL. Test sample solutions (10 μL) and prepared bacterial suspension (90 μL) containing 1 × 10^6^ cfu/mL were added into each well of the 96-well microplate. The negative control well contained 90 μL of the inoculum (1×10^6^ cfu/mL) and 10 μL of 30% acetone. Streptomycin sulfate was used as the positive control. After the plates were agitated to mix the contents of the wells using a plate shaker and incubated in the dark at 28 °C for 24 h, 10 μL of MTT (5 mg/mL in 0.2 mol/L, pH 7.2 phosphate-buffered saline) was added into each well, and the plates were incubated for another 4 h. The minimum inhibitory concentration (MIC) value was defined as the lowest sample concentration that inhibited visible growth, as indicated by the MTT staining. Only living microorganisms could convert MTT to formazan and a blue color appeared in the well [21]. The minimum bactericidal concentration (MBC) was determined as the highest dilution at which no growth occurred in medium. To confirm the MBC, 10 µL of the suspension liquid was removed from each well and inoculated on the LB medium. After aerobic incubation at 28 °C overnight, the number of surviving organisms was determined.

To further determine the median inhibitory concentration (IC_50_) value of the samples, the above microplates incubated with MTT were centrifuged at 1500 g for another 20 min. Then the supernatant was aspirated, 200 μL of dimethyl sulfoxide (DMSO) was added into each well, and the colored formazan products were extracted for 30 min. After complete extraction, the plate was centrifuged at 1,500 g for another 20 min, and then 100 μL of the supernatant (DMSO solution) in each well was transferred to a corresponding well of another 96-well microplate to measure their light absorption values at wavelength 510 nm using a microplate spectrophotometer. The percentage (%) of the bacterial growth inhibition was determined as [(*A*_c_–*A*_t_)/*A*_c_] × 100, where *A*_c_ was an average of six replicates of light absorption values at wavelength 510 nm of the negative controls, and *A*_t_ was an average of six replicates of light absorption values at wavelength 510 nm of the samples. The IC_50_ value was calculated using the linear relation between the inhibitory probability and concentration logarithm according to the method of Sakuma [22]. The IC_50_ value was expressed as the mean ± standard deviation of three independent experiments.

#### 3.6.2. Antifungal activity assay

The dilution-colorimetric assay was employed to investigate the anticandidal activity of the essential oil and 2-hydroxy-4-methoxybenzaldehyde. *Candida albicans* ATCC 10321 was grown in liquid potato dextrose (PD) medium overnight at 28 °C, and the diluted *C. albicans* suspension (10^6^ cfu/mL) was ready for assay. The final concentrations of the samples ranged from 0.05 to 0.30 mg/mL containing 3% (v/v) acetone. Other procedures for determining MIC, minimal fungicidal concentration (MFC) and IC_50_ values were the same as those for antibacterial activity assay described above only amphotericin B used as the positive control and PD medium instead of LB medium.

Rice blast fungus, *Magnaporthe oryzae* (strain P131) was maintained on the oatmeal-tomato agar medium (oatmeal 30 g/L, tomato juice 150 mL/L, and agar 20 g/L) at 25 °C. The spores were prepared from 7-day-old cultures of *M. oryzae*, according to our previous report [23]. The sample-acetone solution (25 μL) was mixed with an equivalent volume of spore suspension containing 2 × 10^6^ spores per mL. The mixture was then placed on separate concave glass slides. The final concentrations of the samples ranged from 0.10 to 0.35 mg/mL containing 5% (v/v) acetone. The negative control was 5% acetone, and the positive control was carbendazim at concentrations ranging from 0.01 to 0.10 mg/mL. Three replicates were used for each treatment. Slides containing the spores were incubated in a moist chamber at 25 °C for 7 h. Each slide was then observed under the microscope for spore germination status. About 100 spores per replicate were observed to detect spore germination. The percentage (%) of spore germination inhibition was determined as [(*G*_c_–*G*_t_)/*G*_c_] × 100, where *G*_c_ is an average of three replicates of germinated spore numbers in the negative control, and *G*_t_ is an average of three replicates of germinated numbers in the treated sets. The IC_50_ value calculation for the spore germination inhibition was the same as that for antibacterial activity assay. The MIC value on the spore germination was defined as the lowest sample concentration that inhibited visible spore germination. The MFC value on the spore germination was determined as the highest dilution at which no spore germination and mycelial growth occurred on the oatmeal-tomato agar medium.

### 3.7. Antioxidant activity

#### 3.7.1. DPPH radical scavenging assay

Radical scavenging assay was determined by a microplate spectrophotometric method based on the reduction of a methanol solution of DPPH using the method of Ono *et al*. [24]. Briefly, DPPH solution (80 μL, 0.2 mg/mL) and essential oil solution in 30% acetone (20 μL) were added to each well of the microplate and mixed. The mixture was shaken vigorously and left to stand at 37 °C for 30 min in the dark. The absorbance of the solution was then measured at wavelength 515 nm using a microplate spectrophotometer. Inhibition (%) of free radical (DPPH) in percent was determined as [(*A*_control_-*A*_sample_)/*A*_control_] × 100, where *A*_control_ is the absorbance of the control reaction containing all reagents except the test sample, and *A*_sample_ is the absorbance of the test oil. Tests were carried out in triplicate. BHT was used as the positive control. The IC_50_ was calculated using linear relation between the essential oil concentration and probability of the percentage of DPPH inhibition.

#### 3.7.2. β-Carotene-linoleic acid bleaching assay

The antioxidant activity of the essential oil was evaluated by β-carotene-linoleic acid bleaching method described by Ebrahimabadi *et al*. [25] with slight modifications. Briefly, linoleic acid (25 μL) and Tween-40 (200 mg) were added in the β-carotene solution (0.5 mg of β-carotene dissolved in 1 mL of chloroform). Chloroform was then removed using a rotary evaporator at 50 °C. Distilled water (50 mL) saturated with oxygen for 30 min at a flow rate of 100 mL/min were added and the mixture was vigorously shaken. The above β-carotene-linoleic acid-Tween mixture (90 μL) and the essential oil solution (10 μL, concentrations from 1.5 mg/mL to 3.0 mg/mL) in 30% acetone solution were added into each well. An equal amount of 30% acetone was used as the control. The microplates were then placed in an incubator at 50 °C for 2 h together with BHT as the positive control. The absorbance of the solution was then measured at wavelength 460 nm using a microplate spectrophotometer. The percentage (%) of β-carotene bleaching inhibition of each sample was determined as (*A*_β -carotene after 2 h assay_/*A*_initial_ _β-carotene_) × 100, Where *A*_β -carotene after 2 h assay_ is the absorbance of the sample with β-carotene-linoleic acid mixture after 2 h period of incubation, and *A*_initial_ _β-carotene_ is the absorbance of the initial mixture. All tests were carried out in triplicate. The IC_50_ value calculation for β-carotene bleaching inhibition was the same as that for antibacterial activity assay.

#### 3.7.3. Metal chelating activity on ferrous ions (Fe^2+^)

Metal chelating activity was determined according to the method of Oke *et al*. [26] with some modifications. Briefly, the essential oil in 30% acetone (20 μL) was mixed with 0.65 mmol/L FeCl_2_ and 60 μL of 4 mmol/L ferrozine disodium salt (20 μL) in each well. Then, the mixture was shaken vigorously and left standing at room temperature for 10 min. After the mixture reached equilibrium, the absorbance of the solution was then measured at wavelength 560 nm using a microplate spectrophotometer. The ferrous ions chelating effect was calculated as the percentage (%) of inhibition of ferrozine-Fe^2+^ complex formation determined as [(*A*_control_-*A*_sample_)/*A*_control_] × 100, Where *A*_control_ is the absorbance of the only ferrozin-Fe^2+^ complex, and *A*_sample_ is the absorbance of the test essential oil and ferrozin-Fe^2+^ mixture. EDTA was used for the positive control. The IC_50_ value calculation for ferrozine-Fe^2+^ complex formation was the same as that for antibacterial activity assay.

## 4. Conclusions

This is the first report on the antimicrobial and antioxidant activities of *P. sepium* essential oil and its main component 2-hydroxy-4-methoxybenzaldehyde. Both of them were screened to show a wide spectrum of inhibitory activity against pathogenic bacteria and fungi, as well as a moderate antioxidant activity. As 2-hydroxy-4-methoxybenzaldehyde was the most abundant component (78.8%), the antimicrobial and antioxidant activity of the root bark essential oil of *P. sepium* could be mainly attributed by this compound. 2-Hydroxy-4-methoxybenzaldehyde has been used as a flavoring agent for the preparation of soft drinks and bakery products [27]. This compound was stably produced in the continuous root culture of *Hemidesmus indicus* (Asclepiadaceae) [28]. Vanillin was also tested to be effective against *Saccharomyces cerevisiae* and *Candida parapsilosis* [29]. Paeonol had multi-activities such as anti-inflammatory, anti-tumor, and anti-atherosclerosis effects [30]. Phenolic compounds including 2-hydroxy-4-methoxybenzaldehyde, paeonol, vanillin and methyl 4-methoxysalicylate accounted for 80.1% of the oil. They should be responsible for biological activities of the oil of *P. sepium*. 

The present study has provided additional data supporting the utilization and development of the root bark essential oil of *P. sepium* as an antimicrobial and antioxidant agent. The essential oil of *P. sepium* could be an alternative resource for preparing 2-hydroxy-4-methoxybenzaldehyde. This study also provided some basal data in aspects of antimicrobial and antioxidant activities for 2-hydroxy-4-methoxybenzaldehyde as an important aroma and flavoring agent in production of soft drinks and bakery products.
